# IL-2 and IL-7 Contribution to Immune Response: Effects of Vaccination Against COVID-19 in Adults

**DOI:** 10.3390/v17111416

**Published:** 2025-10-24

**Authors:** Dominika Siedlecka, Lena Bielawska, Aleksandra Ludziejewska, Aleksandra Baszczuk, Ewa Wysocka

**Affiliations:** Department of Laboratory Diagnostics, Poznan University of Medical Sciences, 60-569 Poznan, Poland

**Keywords:** COVID-19, interleukin-2, interleukin-7, immunity, SARS-CoV-2, vaccine

## Abstract

Background: Cytokines participate in regulating the immune response of lymphocytes. Interleukin 2 (IL-2) is the main modulator of T lymphocyte development, homeostasis, and function, whereas interleukin 7 (IL-7) regulates the development and homeostasis of immune cells and plays a crucial role in the maintenance of memory cells. The study aims to assess the blood IL-2 and IL-7 concentration in relation to the obtained cellular and humoral response in adults, six months after vaccination against COVID-19. Methods: We measured the concentration of IL-2 and IL-7 with ELISA, CoV2-IgG with an indirect chemiluminescence test, and the levels of IFN-γ with interferon gamma release assay (IGRA) post SARS-CoV-2 antigen stimulation. The study group (*n* = 76; F = 66, M = 10) was divided into 41 individuals, who did not report any chronic disorder (ChrD-Neg), and 35, who did (ChrD-Pos). Results: ChrD-Pos group presented higher IL-7 compared to ChrD-Neg (*p* = 0.023). Negative correlations were observed in the entire study population between IL-2 and age (R = −0.252, *p* = 0.028), as well as between IL-7 and IFN-γ (R = −0.295, *p* = 0.010). We found a positive correlation between IL-2 and IL-7 concentrations in the entire study population (R = 0.305, *p* = 0.007) and the ChrD-Pos group (R = 0.358, *p* = 0.035), and people with a positive IGRA result (R = 0.359, *p* = 0.005). Conclusions: The interaction of IL-2 and IL-7 may be important for achieving post-vaccination immunity, especially in adults with chronic diseases. Age is a factor modifying the post-vaccination response (decreased IL-2), whereas IL-7 may be an important factor in achieving a satisfactory post-vaccine response in people with chronic diseases.

## 1. Introduction

The severe acute respiratory syndrome coronavirus 2 (SARS-CoV-2) is the etiological agent of the coronavirus disease 2019 (COVID-19), which has resulted in a pandemic across the globe and a tremendous loss of human life [1]. The first COVID-19 case was reported in China in December 2019. The COVID-19 pandemic was officially declared by the World Health Organization (WHO) on 11 March 2020, following the spread of epidemic outbreaks around the world. It was possible to announce the end to the pandemic on 5 May 2023, because of the rise in population immunity brought about by diseases and vaccines [2].

Active immunity is of two types: naturally acquired following infection and vaccine-induced immunity [3,4]. The European Commission approved the following COVID-19 vaccine types: mRNA, vector, protein, and inactivated whole virus vaccines. This followed the EMA’s positive scientific opinions. In time, though, the approval of the COVID-19 Vaccine Jcovden, Valneva, Vaxzevria, and VidPrevtyn Beta has been withdrawn [5,6,7,8,9,10,11,12]. At least one dose of the COVID-19 vaccine has been administered to 70.6% of the global population. A total of 13.57 billion doses have been administered globally, while these days, 2631 doses are given per day [13].

The immune system gets activated when the upper respiratory tract comes in contact with the SARS-CoV-2 virus. The virus binds to angiotensin-converting enzyme 2 (ACE2), which is a receptor for SARS-CoV-2 and mediates the entry of the virus into host cells, through spike (S) glycoproteins found on the viral outer surface. COVID-19 induces both innate and adaptive immune responses. Cellular immunity is developed by CD8+ T cells, which infect and destroy virus-infected cells, and humoral immunity by CD4+ T cells, stimulating B cells to produce and secrete anti-SARS-CoV-2 antibodies (immunoglobulin, Ig) [14,15,16]. After an infection occurs, S-specific IgM, IgA, and IgG antibodies are identified. The levels of IgG antibodies (CoV2-IgG) and the presence of IgG memory B cells can remain for an extended period following an infection or immunization [17,18]. IFN-γ is a cytokine produced by natural killer (NK) cells as an element of the innate immune response and CD4+ and CD8+ T cells after developing specific immunity [19]. The cytokine could also be measured when released from T cells during a laboratory test (interferon gamma release assay, IGRA).

During the immune response, the level of interleukin changes. Interleukins are cytokines that participate in interactions between innate and adaptive immunity. They are produced mainly by leukocytes, but fibroblasts, adipocytes, and endothelial cells also have the ability to produce them. For example, interleukin 2 (IL-2) is secreted by T lymphocytes, while bone marrow stromal cells produce interleukin 7 (IL-7) [20,21]. IL-2 is the main modulator of T lymphocyte development, homeostasis, and function. It acts as a molecular bridge between the innate and adaptive immune response cells, and is important in the activation of T cells following primary activation and reactivation of memory T cells. IL-2 is a key regulator of T cell biology. In the thymus, IL-2 is responsible for the proliferation of naive lymphocytes and is also essential for the maturation of Tregs. It is also responsible for the expansion and cytotoxicity of effector T cells. The primary producers of IL-2 are effector T cells. IL-2 is used to stimulate their own proliferation, cytotoxicity, and the further development of memory T cells. IL-2 levels influence the expansion, maturation, activity, and cytotoxicity of NK cells. IL-2 also affects B lymphocytes, stimulating their proliferation and supporting antibody production [22,23]. IL-7 regulates the development and homeostasis of immune cells, including T lymphocytes, B lymphocytes, and NK cells. It plays a crucial role in the maintenance of health and prevention of disease. Increased IL-7 production promotes the survival of both naive and memory T cells. It is a cytokine that can regulate the recruitment of leukocytes, such as neutrophils and monocytes. IL-7 is essential for the development of murine B cells, where it is responsible for their survival, differentiation, and proliferation. However, unlike in mice, human B cell development could be observed in the absence of IL-7. Research indicates that IL-7 also plays a role in the maturation of NK cells and plays a role in their homeostasis in the spleen. The lack of IL-7 causes the retention of immature immune cells [24,25].

**Figure 1:**
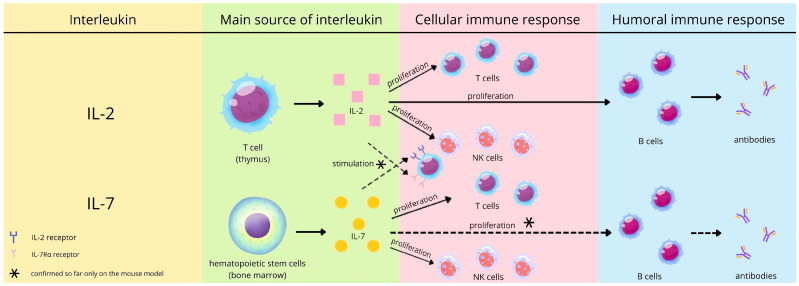
The expected role of interleukin 2 and 7 in the immune response (prepared based on 22–27).

Dooms et al., analyzing the mouse model, proved that IL-2 enhances CD4+ T cell memory by promoting the generation of IL-7Rα-expressing cells [26]. Zhang et al. also studied a mouse model and proved that IL-7 significantly induced the expression of the IL-2 receptor [27]. Moreover, the results of the research by Coppola et al. suggest a synergistic interaction of IL-2 and IL-7 in promoting optimal proliferation and survival of activated CD4+ T lymphocytes [28]. Currently, we have no confirmation that the above-mentioned mechanism occurs in humans and could determine the effectiveness of the post-vaccination response in viral diseases, including COVID-19. Figure 1 illustrates the recognized effects of interleukin 2 and 7 on cellular and humoral immune responses in adults and possible results translated from studies on murine cells.

The study aimed to assess the blood IL-2 and IL-7 concentration in relation to the obtained cellular and humoral response in adults six months after vaccination against COVID-19.

## 2. Materials and Methods

### 2.1. Study Population

The study was conducted on adult Caucasian employees of the University Hospital in Poznan, people aged 19–69, six months after receiving the complete COVID-19 mRNA vaccine (Comirnaty). A COVID-19 vaccination was administered in January or February of 2021. Participants received their second dose of the vaccine 21 days following the first.

Exclusion criteria included vaccination with a vaccine other than Comirnaty, an acute state, and declared sick leave within six months prior to vaccination. One of the basic conditions for participation in the study was the inclusion to the research of professionally active workers who were free from acute symptoms at the time of qualification and blood sampling. We attempted to eliminate the influence of the acute phase on IL-2 and IL-7 concentrations to achieve relatively constant, stable interleukin concentrations.

One hundred ninety-five participants were initially qualified for the study, but one hundred nineteen of them were excluded. Finally, 76 adults (66 females and 10 males) were included in the study.

Of the people who participated in the study, 41 did not report any chronic disorder, and 35 did. Some of the respondents declare more than one chronic disease. Clinical conditions that were taken into account were cardiovascular, endocrine, autoimmune, respiratory, metabolic, oncological, connective tissue, and liver diseases.

The study was conducted in accordance with the Declaration of Helsinki and approved by the Bioethics Committee of Poznan University of Medical Science (protocol code 189/21) on 11 March 2021 for studies involving humans.

### 2.2. Measurements and Analysis

Following current guidelines, whole blood was drawn into a clotting activator (4.9 mL) for serum and a heparin tube (4.9 mL) for whole heparin blood and heparin plasma (S Monovette^®^, Sarstedt, Nümbrecht, Germany).

Through indirect chemiluminescence of the individual’s serum concentration of IgG antibodies to the receptor-binding domain (RBD), a subunit 1 of the viral spike protein (S1) of SARS-CoV-2 was determined. Anti-SARS-CoV-2 IgG antibody level was measured on an Atellica^®^ IM analyzer (Siemens Healthcare Diagnostics Inc., Malvern, PA, USA). The test’s sensitivity was established for material obtained after a specific number of days following a positive PCR test result, and it was as follows: 0–6 days after PCR—50.82%; 7–13 days after PCR—82.47%; 14–20 days after PCR—91.14%; and ≥20 days after PCR—96.41%. Specificity has been established from samples collected before the COVID-19 outbreak, which was estimated to be 99.90%. The test results are reported as an index value with the designation of non-reactive or reactive: non-reactive: index value < 1.00 (no antibodies present); reactive: index value ≥ 1.00 (presence of antibodies) [29].

Whole heparin blood was intended for the IGRA, and heparin plasma was used for determining the concentration of interleukin 2 and 7 using the ELISA test.

The interferon gamma release assay was performed using the IGRA test—Quan-T-Cell-SARS-CoV-2 (Euroimmun, Lübeck, Germany). The IGRA test consisted of two stages: stimulation of T lymphocytes with the SARS-CoV-2-specific S1 domain of the S protein and determination of the concentration of IFN-γ released by T lymphocytes using an ELISA test. The BLANK, STIM, and TEST tubes were used at the first stage of the study. The BLANK tube was used to assess the individual IFN-γ background. This tube did not contain any component that could activate immune cells. The STIM tube was coated with a mitogen that caused a non-specific release of interferon gamma. Based on the significantly higher IFN-γ concentration in the STIM tube than the value obtained from the BLANK tube allowed for verification of the effectiveness of cellular immunity among participants. All study participants had an adequate number of immune cells capable of activation. The TEST tube contains components of the S1 domain of the S protein of the SARS-CoV-2 virus. The IFN-γ concentration taken into account in the statistical analysis is the level of IFN-γ obtained from the TEST tube, reduced by the concentration obtained from the BLANK tube. The results were read using a TECAN microplate reader (Tecan Group Ltd., Männedorf, Switzerland). The test’s specificity was 97% and the test sensitivity was 96%. The interpretation of the IGRA results was according to the recommended ranges from EUROIMMUN: negative: IFN-γ < 100 mlU/mL; borderline: IFN-γ 100–200 mlU/mL; positive: IFN-γ > 200 mlU/mL.

The Human IL2 and IL7 ELISA Kits (Biorbyt, Cambridge, UK) were used for IL-2 and IL-7 determination. The concentration of both interleukins was measured using the TECAN microplate reader (Tecan Group Ltd., Männedorf, Switzerland). The intra- and interassay coefficient of variation (CV) was 4.1–6.2% and 4.1–8.1% for IL-2 and 4.7–6.9% and 5.5–6.9% for IL-7, respectively.

### 2.3. Statistical Methods

PQStat Software v.1.8.4 (Poznan, Poland) and Statistica 13.3 (StatSoft Inc., Tulsa, OK, USA) were the tools used for the statistical analysis. The normality of the data distribution was confirmed using the Shapiro–Wilk test. Non-parametric tests were employed when a normal distribution was not found. All results were expressed using the interquartile range and median. The significance of the differences between the two groups was evaluated using the Mann–Whitney U test, and the Kruskal–Wallis test with Post Hoc analysis of multiple comparisons was used to compare several groups. Spearman’s R coefficient was used to evaluate correlations between the variables under study.

## 3. Results

Table 1 displays the characteristics of the study participants. The statistical analysis of CoV2-IgG, IFN-γ, IL-2, and IL-7 was performed in the entire study population (*n* = 76), the groups not declaring (ChrD-Neg) and reporting chronic diseases (ChrD-Pos) as well. The results are presented in Table 2 with the *p*-value from the Mann–Whitney test. Moreover, the presented parameters did not differ between females and males in the study population. The CoV2-IgG were present in the serum of all tested people, but only 61 were positive for IGRA.

**Figure 2:**
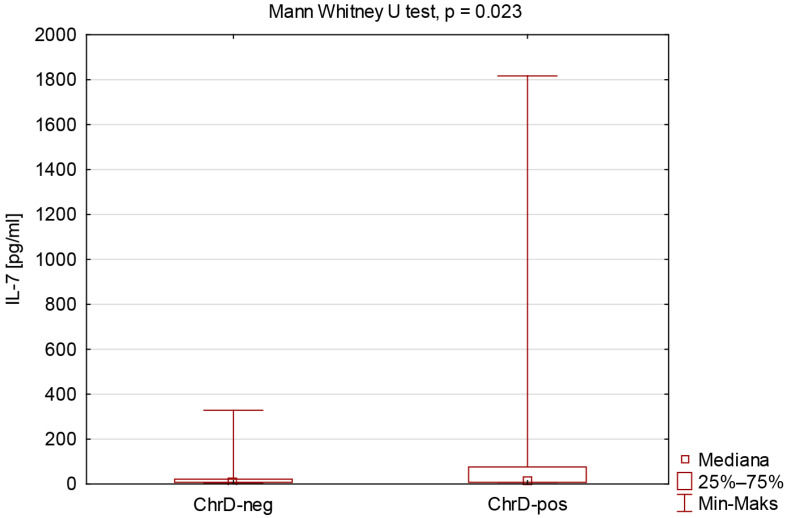
The comparison of IL-7 concentration due to reported chronic diseases.

The comparison of IL-7 concentration due to reported chronic diseases is presented in Figure 2.

The results of the correlation analysis between IL-2 and IL-7 versus age, BMI, CoV2-IgG, IFN-γ, and the mutual relationship of IL-2 and IL-7 in the study groups and subgroups are presented in Table 3.

Table 4 shows the characteristics of the tested parameters and their comparison, according to the levels of IGRA results.

Several increased triple-digit values of interleukins IL-2 and IL-7 were found in the study population. Individuals with such results become distinct when dividing the study population into the IGRA-Negative (elevated IL-2) and the IGRA-Borderline (elevated IL-7) groups. This may be due to the fact that the body attempted to force a post-vaccination response, but insufficiently. It is possible that these individuals had antibodies but lacked immunological memory.

The results of the correlation analysis between IL-2 and IL-7 versus age, BMI, CoV2-IgG, and the mutual relationship of IL-2 and IL-7 in the study groups with respect to the levels of IGRA results are presented in Table 5.

**Figure 3:**
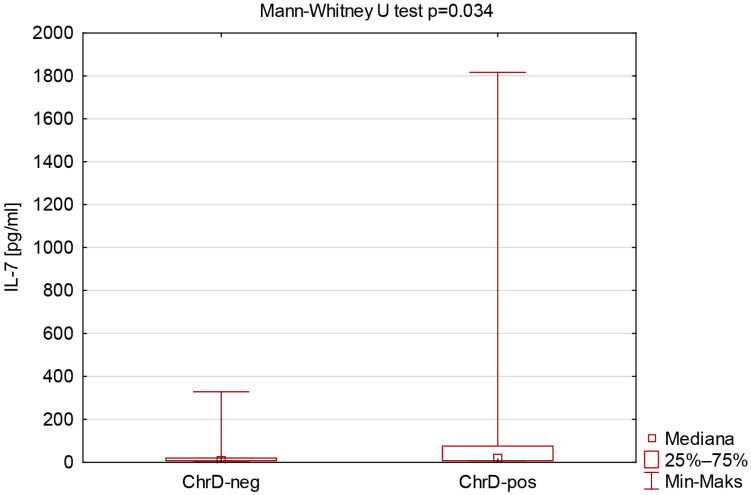
The comparison of IL-7 concentration due to reported chronic diseases in the IGRA-Positive group.

Similar analyses were carried out due to reported chronic diseases in the IGRA-Positive group (Table 6 and Table 7). The comparison of IL-7 concentration due to reported chronic diseases in the IGRA-Positive group is presented in Figure 3.

## 4. Discussion

SARS-CoV-2 infection has revealed several important immunopathological mechanisms of COVID-19, such as the role of cytokines in regulating the lymphocyte response. Two of them, interleukin 2 and interleukin 7, are of significance due to their roles in T lymphocyte biology. IL-2 contributes to T lymphocyte activation, proliferation, and survival, whereas IL-7 is responsible for the maintenance of memory cells [23,30]. The study aimed to assess the blood IL-2 and IL-7 concentration in relation to the obtained cellular and humoral response in adults six months after vaccination against COVID-19.

We compared two groups in our research: individuals not declaring chronic diseases (ChrD-Neg) and professionally active workers reporting a clinical condition (ChrD-Pos), including cardiovascular, endocrine, autoimmune, respiratory, metabolic, oncological, connective tissue, and liver diseases. Both groups achieved similar cellular and humoral responses after vaccination. We determined that the ChrD-Pos group had elevated IL-7 levels.

Despite the fact that the subject of research is IL-2 and IL-7 concentrations, there are few publications dealing with the post-vaccination response against COVID-19. It is worth mentioning that IL-7 is a subject of interest among researchers, with its use as an adjuvant in the development of anti-cancer and anti-HIV vaccines [31,32]. Ojalvo et al. [33] found increased IL-2 levels after COVID-19 vaccination, while Zhu et al. [34] documented increased IL-7 levels. Unfortunately, they do not address the issue of the dependence of their concentrations on age or BMI.

There are certain articles providing data on changes in the concentration of IL-7 based on the presence of specific chronic diseases. Elevated levels of IL-7 in patients with such disorders are supported by results of Damås et al. [35], Bikker et al. [36], and Seyfarth [37]. Damås et al. studied patients with stable (*n* = 30) and unstable angina (*n* = 30) and compared them with healthy control subjects (*n* = 20). Their research had indicated increased plasma IL-7 concentrations [35]. Similarly, Bikker et al. had a review highlighting the pathologic role of IL-7 in autoimmune diseases like rheumatoid arthritis, systemic lupus erythematosus (SLE), multiple sclerosis, and type 1 diabetes, where the concentration of IL-7 was increased [36]. In addition, Seyfarth et al. measured serum IL-7 levels in cystic fibrosis patients (*n* = 164, *n* = 78 for the second time point) and healthy controls (*n* = 60). The results indicated elevated serum IL-7 levels in cystic fibrosis patients that were correlated with chronic decline of lung function [37]. These findings suggest that chronic disease might lead to prolonged elevation of IL-7 concentrations reflecting ongoing immune activation. Reproducibility of our results with previously published research highlights the potential of IL-7 as a marker for chronic inflammation.

Spearman’s correlation test between age and IL-2 concentration in all study groups confirmed a negative correlation (R = −0.252, *p* = 0.028), although it was statistically weak—a stronger correlation could be achieved using the results collected from a larger number of volunteers (in our case, *n* = 76). A similar result was obtained in the IGRA-Negative group (R = −0.868, *p* = 0.005). There are a few studies examining the relationship between IL-2 levels and age. Recent articles focus mainly on the role of IL-2 in immune response and cancer rather than its relationship with aging.

Pahlavani and Richardson found that the age-related decline in IL-2 production is due to a decline in IL-2 transcription [38]. Similar results have been reported by Rea et al., who demonstrated that levels of IL-2 were very low in people in their eighties and nineties [39]. In the context of the COVID-19 pandemic, these results are particularly relevant. The immunosenescence process is characterized by reduced T cell function, limited proliferative capacity, and reduced production of cytokines, including IL-2. Elderly individuals, who physiologically have reduced IL-2 levels, may demonstrate a limited ability to activate and proliferate T cells in response to SARS-CoV-2 infection [40,41]. This may partially explain the greater susceptibility of this age group to a more severe course of the disease and a weaker post-vaccination response. The possibility that IL-2 is a marker of immunosenescence suggests the need for further research on the age-dependent changes in IL-2 levels.

Although among individuals with stable chronic diseases we determined elevated IL-7 levels, our participants did not differ in IL-2 concentrations. Maybe that is why these subgroups did not differ in IFN-γ and CoV2-IgG concentrations. However, we documented a negative correlation of IL-2 versus age, and the absence or presence of chronic disease had no statistical effect.

It would also be appropriate to mention that the performed analyses revealed a negative correlation between IL-7 concentration and IFN-γ level (R = −0.295, *p* = 0.010). Scientific reports indicate that IL-7 plays a key role in maintaining T lymphocyte homeostasis and may modulate their ability to produce cytokines, including IFN-γ. In vitro studies have shown that IL-7 increases IFN-γ expression by affecting the transcription and stability of the mRNA encoding this cytokine [42]. In our study, IFN-γ concentration was obtained after vaccination and proves the efficiency of the cellular response by checking the reactivity of T lymphocytes. The negative correlation applies to six months after vaccination against COVID-19. Further research is needed to elucidate the molecular basis of the relationship between IL-7 and IFN-γ and its potential consequences for the immune response in various clinical conditions.

The analysis of our results demonstrated a positive correlation between IL-2 and IL-7 concentrations observed both in the entire study group and in subgroups (people declaring chronic diseases, individuals with a positive IGRA result). Similar observations were reported by Okazaki et al., who showed that IL-7 induces thymocyte proliferation in vitro, and this effect is significantly enhanced in the presence of IL-2. These data indicate synergy between both cytokines in the maturation and maintenance of T lymphocytes, which may explain the observed correlation in their concentrations in adults successfully vaccinated against COVID-19 [43].

Katzman et al. noted that both cytokines have partially opposing functions in regulating the immune response. While IL-2 promotes the activation and differentiation of effector lymphocytes and plays a key role in the development of regulatory cells, IL-7 is primarily responsible for maintaining T cell survival and homeostasis. The authors suggest that a regulation between IL-2 and IL-7 is essential for proper control of the immune response [23].

This correlation might imply that the simultaneous regulation of these cytokines enhances both the effector phase and long-term immune memory maintenance. This is particularly important because an effective vaccine, in addition to inducing the immediate surge in T cell activity, must provide lasting immunity, for which IL-7 appears to be pivotal. Understanding of this interaction can have therapeutic potential for the design of new immunomodulatory strategies, especially in chronic disease or immune deficiency patients, for whom the homeostasis between IL-2 and IL-7 might be compromised.

The interaction of IL-2 and IL-7 may be important for achieving post-vaccination immunity, especially in adults with chronic diseases. Age may contribute to a decreasing IL-2 concentration. However, reduced lymphocyte reactivity to SARS-CoV-2 stimulus (IFN-γ results) may necessitate IL-7 mobilization. It is possible that increased IL-7 concentration promotes the effectiveness of the COVID-19 vaccination response despite having a history of chronic disease.

Further study on the contribution of IL-2 and IL-7 to immune response effects of vaccination against COVID-19 is needed.

## 5. Limitations of the Study

Some limitations of our study might be considered. The study group is relatively small, and therefore, the number of participants in the subgroups. Because of that, the power of statistical tests was significant, but weak. The study may also be limited by the lack of information about levels of IL-2, IL-7, CoV2-IgG, and IFN-γ before vaccination.

## 6. Conclusions

Our findings highlight that the interaction of IL-2 and IL-7 may be important for achieving post-vaccination immunity, especially in adults with chronic diseases. Age is a factor modifying the post-vaccination response (decreased IL-2), whereas IL-7 may be an important factor in achieving a satisfactory post-vaccine response in people with chronic diseases.

Moreover, IL-2 and IL-7 results in relation to cellular and humoral response six months after vaccination in adults may be used to conduct future meta-analyses and create strategies for dealing with possible future pandemics.

Chronic diseases are and will remain a fundamental medical problem. They determine the actions of modern medicine and will be a growing challenge for doctors in the future. We hope that the results of our study will encourage the evaluation of satisfactory post-vaccination responses among people with chronic diseases.

## Figures and Tables

**Table 1:** The characteristics of the study participants.

**Parameter**	**All** ***n* = 76**	
Age [years]	Me (Q1–Q3)	46.50 (34.00–57.00)
	mean ± SD	44.5 ± 13.5
	min–max	19.0–69.0
Gender [F/M]	66/10	
BMI [kg/m^2^]	Me (Q1–Q3)	24.57 (22.25–27.63)
	mean ± SD	25.41 ± 4.60
	min–max	17.58–42.52
**Category** **of chronic disease**	**Number of study participants [*n*]**	
Cardiovascular	11	
Endocrine	9	
Autoimmune	9	
Respiratory	8	
Metabolic	5	
Oncological	2	
Connective tissue	1	
Liver	1	
**Set of chronic disease**	**Number of study participants [*n*]**	
1 disease	25	
2 diseases	8	
3 diseases	1	
4 diseases	1	

**Table 2:** The comparison of age, BMI, CoV2-IgG, IFN-γ, IL-2, and IL-7 due to reported chronic diseases.

Parameter	All *n* = 76	ChrD-Neg *n* = 41	ChrD-Pos *n* = 35	*p* Mann–Whitney Test
Age [years]	46.50 (34.00–57.00)	45.00 (31.00–52.00)	47.00 (36.00–58.00)	0.18
BMI [kg/m^2^]	24.57 (22.25–27.63)	25.03 (22.31–27.55)	24.24 (22.10–27.68)	0.95
CoV2-IgG [U/mL]	9.15 (5.00–19.75)	9.40 (5.10–19.60)	8.50 (4.80–20.50)	0.62
IFN-γ [mIU/mL]	553.53 (257.77–1287.52)	619.56 (323.14–1293.61)	494.96 (176.14–1017.70)	0.29
IL-2 [pg/mL]	15.60 (15.60–95.91)	15.60 (15.60–104.79)	15.60 (15.60–64.11)	0.93
IL-7 [pg/mL]	7.80 (7.80–47.67)	7.80 (7.80–21.84)	11.53 (7.80–76.07)	**0.023** *

Data are presented as median and interquartile range: Me (Q1–Q3). * Statistically significant results.

**Table 3:** The correlations of the analyzed parameters in the study group and subgroups due to reported chronic diseases.

Parameters	All *n* = 76	ChrD-Neg *n* = 41	ChrD-Pos *n* = 35
IL-2 and age	**R = −0.252, *p* = 0.028** *	R = −0.259, *p* = 0.10	R = −0.264, *p* = 0.13
IL-2 and BMI	R = 0.036, *p* = 0.76	R = 0.180, *p* = 0.26	R = −0.145, *p* = 0.41
IL-2 and CoV2-IgG	R = 0.128, *p* = 0.27	R = 0.252, *p* = 0.11	R = −0.005, *p* = 0.98
IL-2 and IFN-γ	R = −0.075, *p* = 0.52	R = −0.137, *p* = 0.39	R = 0.025, *p* = 0.89
IL-7 and age	R = −0.101, *p* = 0.38	R = −0.059, *p* = 0.72	R = −0.240, *p* = 0.16
IL-7 and BMI	R = 0.062, *p* = 0.60	R = 0.218, *p* = 0.17	R = −0.056, *p* = 0.75
IL-7 and CoV2-IgG	R = −0.065, *p* = 0.58	R = −0.143, *p* = 0.37	R = 0.043, *p* = 0.81
IL-7 and IFN-γ	**R = −0.295, *p* = 0.010** *	R = −0.213, *p* = 0.18	R = −0.297, *p* = 0.08
IL-2 and IL-7	**R = 0.305, *p* = 0.007** *	R = 0.273, *p* = 0.08	**R = 0.358, *p* = 0.035** *

* Statistically significant results, CoV2-IgG—anti SARS-CoV-2 IgG.

**Table 4:** The comparison of age, BMI, CoV2-IgG, IL-2, and IL-7 due to the levels of IGRA results.

Parameter	IGRA-Negative *n* = 8	IGRA-Borderline *n* = 7	IGRA-Positive *n* = 61	*p* Kruskal–Wallis Test
Age [years]	55.00 (40.50–61.00)	50.00 (38.00–59.00)	45.00 (31.00–55.00)	a, c 1.00 b 0.34
BMI [kg/m^2^]	25.73 (22.21–28.92)	24.61 (22.60–27.34)	24.52 (22.23–27.68)	a, b, c 1.00
CoV2-IgG [U/mL]	11.60 (6.75–16.10)	6.20 (1.30–13.90)	9.30 (5.00–23.50)	a 0.56 b 1.00 c 0.20
IL-2 [pg/mL]	75.09 (15.60–359.39)	15.60 (15.60–34.80)	15.60 (15.60–80.35)	a 0.76 b 0.87 c 1.00
IL-7 [pg/mL]	11.72 (9.23–24.24)	30.00 (7.80–228.43)	7.80 (7.80–47.89)	a, b 1.00 c 0.43

Data are presented as median and interquartile range: Me (Q1–Q3). a, *p*-value between the IGRA-negative and the IGRA-borderline. b, *p*-value between the IGRA-negative and the IGRA-positive. c, *p*-value between the IGRA-borderline and the IGRA-positive.

**Table 5:** The correlations in the study groups with respect to the levels of IGRA results.

Parameters	IGRA-Negative *n* = 8	IGRA-Borderline *n* = 7	IGRA-Positive *n* = 61
IL-2 and age	**R = −0.868, *p* = 0.005** *	R = −0.022, *p* = 0.96	R = −0.208, *p* = 0.11
IL-2 and BMI	R = 0.241, *p* = 0.57	R = 0.445, *p* = 0.32	R = −0.022, *p* = 0.86
IL-2 and CoV2-IgG	R = 0.355, *p* = 0.39	R = −0.668, *p* = 0.10	R = 0.119, *p* = 0.36
IL-7 and age	R = −0.024, *p* = 0.95	R = 0.736, *p* = 0.06	R = −0.218, *p* = 0.09
IL-7 and BMI	R = 0.491, *p* = 0.22	R = 0.126, *p* = 0.79	R = 0.011, *p* = 0.93
IL-7 and CoV2-IgG	R = 0.132, *p* = 0.76	R = 0.090, *p* = 0.85	R = −0.038, *p* = 0.77
IL-2 and IL-7	R = −0.274, *p* = 0.51	R = 0.360, *p* = 0.43	**R = 0.359, *p* = 0.005** *

* Statistically significant results, CoV2-IgG—anti SARS-CoV-2 IgG.

**Table 6:** The comparison of age, BMI, CoV2-IgG, IL-2, and IL-7 due to reported chronic diseases in the IGRA-Positive group.

Parameter	IGRA-Positive *n* = 61		*p* Mann–Whitney Test
	ChrD-Neg *n* = 35	ChrD-Pos *n* = 26	
Age [years]	43.00 (26.00–54.00)	45.50 (36.00–57.00)	0.37
BMI [kg/m^2^]	25.03 (22.23–27.34)	24.13 (21.37–27.77)	0.95
CoV2-IgG [U/mL]	9.30 (4.70–21.40)	8.95 (5.20–25.50)	0.76
IL-2 [pg/mL]	15.60 (15.60–97.99)	15.60 (15.60–64.11)	0.71
IL-7 [pg/mL]	7.80 (7.80–20.15)	16.84 (7.80–76.07)	**0.034** *

Data are presented as median and interquartile range: Me (Q1–Q3). * Statistically significant results.

**Table 7:** The correlations due to reported chronic diseases in the IGRA-Positive group.

Parameters	IGRA-Positive *n* = 61	
	ChrD-Neg *n* = 35	ChrD-Pos *n* = 26
IL-2 and age	R = −0.261, *p* = 0.13	R = −0.139, *p* = 0.50
IL-2 and BMI	R = 0.151, *p* = 0.39	R = −0.233, *p* = 0.25
IL-2 and CoV2-IgG	R = 0.217, *p* = 0.21	R = −0.007, *p* = 0.97
IL-7 and age	R = −0.119, *p* = 0.50	**R = −0.444, *p* = 0.023** *
IL-7 and BMI	R = 0.190, *p* = 0.27	R = −0.124, *p* = 0.54
IL-7 and CoV2-IgG	R = −0.177, *p* = 0.31	R = 0.084, *p* = 0.68
IL-2 and IL-7	R = 0.315, *p* = 0.07	**R = 0.398, *p* = 0.044** *

* Statistically significant results, CoV2-IgG—anti SARS-CoV-2 IgG.

## Data Availability

The raw data supporting the conclusions of this article will be made available by the authors on request.

## Abbreviations

The following abbreviations are used in this manuscript:

ACE2	Angiotensin-Converting Enzyme 2
COVID-19	Coronavirus Disease 2019
IGRA	Interferon Gamma Release Assay
Ig	Immunoglobulin
IL-2	Interleukin 2
IL-7	Interleukin 7
NK	Natural Killer
RBD	Receptor-Binding Domain
SARS-CoV-2	Severe Acute Respiratory Syndrome Coronavirus 2
S1	Subunit 1 of the Viral Spike Protein
WHO	World Health Organization
